# Early budget impact analysis on magnetic seed localization for non-palpable breast cancer surgery

**DOI:** 10.1371/journal.pone.0232690

**Published:** 2020-05-13

**Authors:** Melanie Lindenberg, Anne van Beek, Valesca Retèl, Frederieke van Duijnhoven, Wim van Harten

**Affiliations:** 1 Division of Psychosocial Research and Epidemiology, The Netherlands Cancer Institute—Antoni van Leeuwenhoek, Amsterdam, The Netherlands; 2 Department of Health Technology and Services research, University of Twente, Enschede, The Netherlands; 3 Division of Surgical Oncology, The Netherlands Cancer Institute—Antoni van Leeuwenhoek, Amsterdam, The Netherlands; Mayo Clinic, UNITED STATES

## Abstract

**Introduction:**

Current localization techniques used in breast conserving surgery for non-palpable tumors show several disadvantages. Magnetic Seed Localization (MSL) is an innovative localization technique aiming to overcome these disadvantages. This study evaluated the expected budget impact of adopting MSL compared to standard of care.

**Methods:**

Standard of care with Wire-Guided Localization (WGL) and Radioactive Seed Localization (RSL) use was compared with a future situation gradually adopting MSL next to RSL or WGL from a Dutch national perspective over 5 years (2017–2022). The intervention costs for WGL, RSL and MSL and the implementation costs for RSL and MSL were evaluated using activity-based costing in eight Dutch hospitals. Based on available list prices the price of the magnetic seed was ranged €100-€500.

**Results:**

The intervention costs for WGL, RSL and MSL were respectively: €2,617, €2,834 and €2,662 per patient and implementation costs were €2,974 and €26,826 for MSL and RSL respectively. For standard of care the budget impact increased from €14.7m to €16.9m. Inclusion of MSL with a seed price of €100 showed a budget impact of €16.7m. Above a price of €178 the budget impact increased for adoption of MSL, rising to €17.6m when priced at €500.

**Conclusion:**

MSL could be a cost-efficient localization technique in resecting non-palpable tumors in the Netherlands. The online calculation model can inform adoption decisions internationally. When determining retail price of the magnetic seed, cost-effectiveness should be considered.

**Highlights**

- Magnetic Seed Localization could be a cost-efficient alternative to RSL and WGL
- In determining the price of MSL, cost-effectiveness should be considered
- The online calculation model can inform adoption decisions on an institutional level

## Introduction

Breast-conserving surgery (BCS) for non-palpable tumors requires appropriate localization technologies to resect the malignancy effectively [1,2]. Currently, mainly two localization technologies are used in the Netherlands: Wire-Guided Localization (WGL) and Radioactive Seed Localization (RSL). RSL aimed to overcome challenges in the use of WGL: challenging hospital planning, potential wire migration, and unfavorable incision placement [3,4]. RSL was shown to be at least non-inferior to WGL on important outcome measures such as re-excision rates and positive surgical margins [5–9]. Moreover, in some studies, RSL has demonstrated improved patient convenience [10–12] and greater ease of use during surgery [12].

RSL however has a considerable disadvantage as its radioactive nature requires adherence to strict nuclear safety regulations [13,14]. This results in a complex implementation process and substantial upfront costs which may explain the relative slow adoption of RSL. In addition, the treatment process may be affected due to the time limitation for an iodine seed to remain in situ (e.g. in the US). To overcome these challenges but retain the advantages of RSL, non-radioactive technologies such as Magnetic Seed Localization (MSL) have been developed. MSL has been shown to be safe and effective in localizing and excising non-palpable breast tumors [15,16]. Therefore MSL seems to be a realistic alternative for RSL and WGL.

A recent study compared WGL with MSL and concluded that WGL was equally effective as MSL[17]. Several studies have shown that RSL is not superior to WGL in clinical outcomes, [5,18]. Therefore, it has been hypothesized that its effectiveness is similar to that of RSL and WGL. When the effectiveness of all three localization modalities are comparable, the widespread adoption of MSL depends on superiority on other aspects such as financial impact and usability.

This study aims to inform the adoption decision of MSL by evaluating the financial impact of gradually adopting MSL as a localization technology for guiding breast conserving tumor excision in the Netherlands health system compared to standard of care (SoC) by means of a Budget Impact Analysis (BIA) incorporating treatment and implementation costs. Secondly, a threshold analysis was conducted to estimate the maximum price level for MSL to become the most cost efficient technology. Finally, the BIA model was made available in a tool to enable translation of the results to other countries or specific hospital settings (S4 Appendix).

## Methods

### 2.1 Budget impact analysis

**Fig 1:**
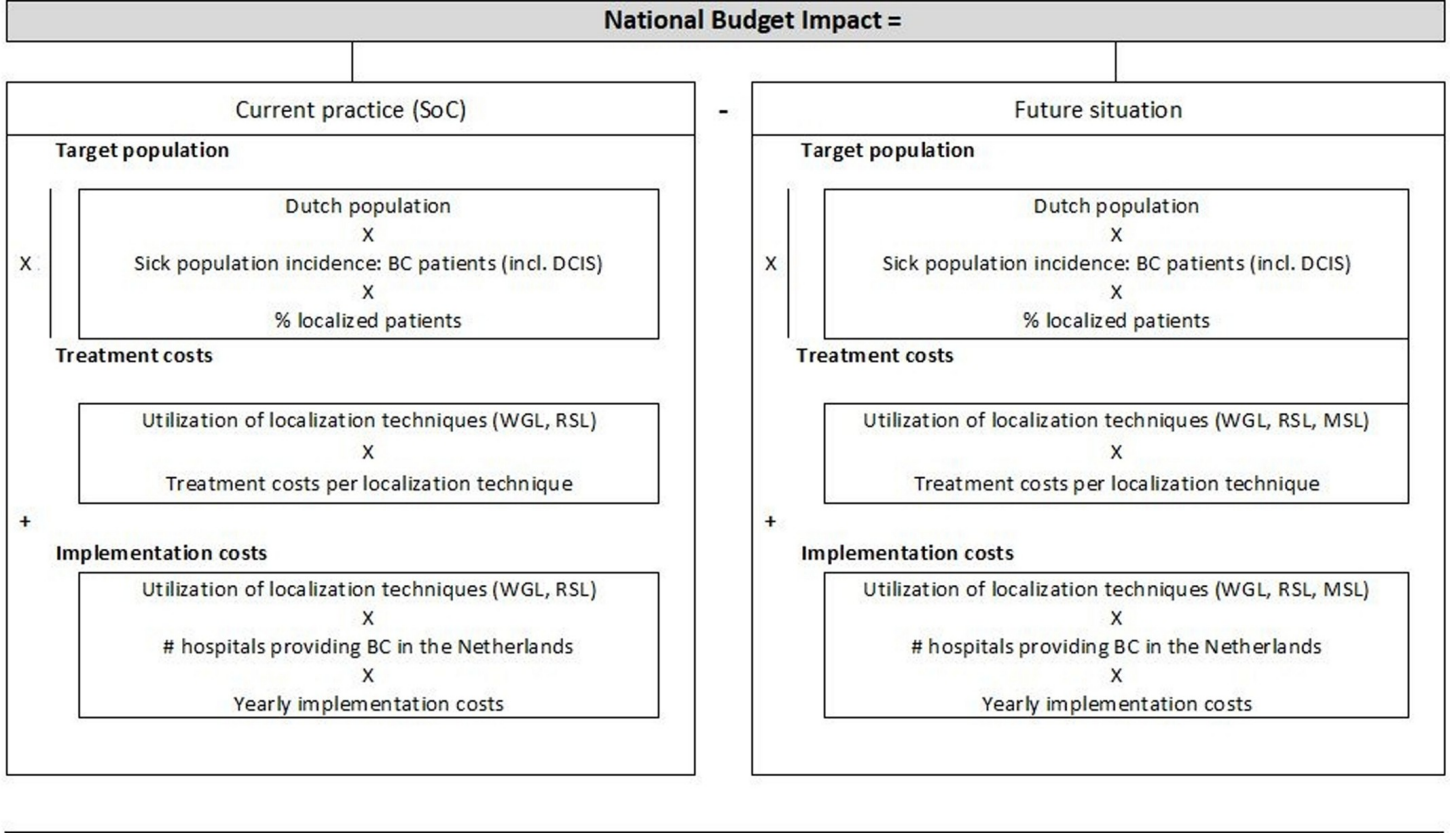
Structure of the Budget Impact model. This national BIA model compares the costs of the current use of localization techniques (RSL and WGL) in the Dutch population with the costs of a future situation in which MSL is adopted. Abbreviations: BC = breast cancer, DCIS = ductal carcinoma in situ, WGL = wire-guided localization, RSL = radioactive seed localization, MSL = magnetic seed localization.

For the analysis, the BIA framework of the International Society for Pharmacoeconomics and Outcomes Research was followed [19]. The analysis was conducted from a Dutch population perspective using a 5-year time horizon (2017–2022). In the Netherlands, RSL and WGL accounted for over 90% of current localization techniques, therefore these were assumed to be the only localization techniques in the current situation [20]. The BI model compares SoC in which both RSL and WGL are used in its present relative “market shares” [20] and a future situation in which MSL is gradually being adopted over time by the Dutch hospitals (Fig 1). The interventions are described in Box 1. Key inputs for the BI model were: size of the target population, utilization of the localization technologies, intervention costs, and the yearly implementation costs for hospitals transferring to either RSL or MSL. The implementation costs were calculated over the first five years that a localization technique is used, starting in the year before its adoption, meaning the first time a technology is used in clinical practice.

**Box 1 - Description of the interventions**

In WGL, a metal wire with a hooked tip is placed in the lesion at the radiology department. This placement needs to be performed on the same day as surgery which complicates scheduling of the surgery. Intraoperatively, the surgeon removes the lesion guided by the wire.[21,22]

With the use of RSL, a small radioactive iodine-125 seed is placed in the lesion by the radiologist. The timing of the placement of the seed in the Netherlands is flexible, and not limited to a few days before surgery. Intraoperatively, a gamma probe providing continuous audible feedback is used by the surgeon to detect the seed. After surgery, the Iodine-125 seed must be removed from the excised specimen in the pathology lab to be safely disposed.[4] We found that using RSL requires an additional availability of staff (e.g. pathology analyst, radiation expert, nuclear medicine staff, RNC assistant) for ~37.5 min per patient compared to WGL. Using RSL also brings the risk of having incidents with radioactive material. From the five hospitals using RSL participating in our study, we identified that each year, on average once or twice a year, the following incidents occur: transection, seed loss, and “near incidents”. A “near incident” is for example a situation in which it is thought to have lost an iodine seed which “needs formal follow-up in view of radioactivity regulations. For this reason the availability of a radiation expert or staff from the nuclear department is required. Transection and seed loss results in at least 2 days of work and “near incidents” in approximately 6 hours.

With the use of MSL, a magnetic seed is placed in the lesion by the radiologist. The signal will not decay over time, therefore the timing of placement is flexible (in feasibility studies the seed has been in situ for a limited period of 2–30 days prior to surgery [15,23]). Intraoperatively, a magnetic probe providing constant feedback on the location of the seed is used by the surgeon to guide resection of the tumor[15,16]. Although the workflow is similar to RSL, no additional activities are required for intake, pathologic analysis and disposal of the seed. S1 Appendix shows the process of each localization technology.

### 2.2 Model inputs

#### 2.2.1 Patient population.

In 2017, 17,207 patients were diagnosed with BC (including DCIS) [24]. The National Institute for Public Health estimates a 15% increase in the incidence of BC over a period of 2015 to 2040 [25]. This increase was assumed to be constant. The number of BC patients receiving localization was estimated on registry data from 2014 [20]. This rate (31.8%) was assumed to be stable over time as no prospective data was available. A proportion (19%) of the target population received neo-adjuvant chemotherapy (NACT) [26]. Receiving NACT was included in the BIA model because it has an influence on the workflow and thus on the intervention costs. When receiving NACT an additional marker is placed for response monitoring when MSL and WGL is used, because the magnetic seed is not compatible with MRI response measurements and the wire can migrate over time. In RSL no additional marker is needed because in the European setting, the iodine seed can be used for response monitoring as the iodine seed is allowed to be in situ for a long period (>30 days). A constant annual increase of 1.3% in receiving NACT was assumed based on historical trends. The input parameters are listed in Table 1.

**Table 1:** Input parameters for the Budget Impact Analysis model.

**Parameter**						**Values**						**Source**	
**Patient population**													
**Breast cancer incidence in the Netherlands:**													
2017 2018 2019 2020 2021 2022						17207 17308 17409 17510 17611 17712						Dutch registries [24,25]	
**Percentage of these BC patients:**													
that receives localization (%)						31.8%						[20]	
that receives neo-adjuvant chemotherapy (%) annual increase of % receiving NACT # Hospitals that provide BC care in 2017						19% 1.3% 105						[26] Based on historic trends [20,26] [27]	
**Future utilization of localization techniques in Dutch patients** *Standard of care scenario (without MSL)* • Assumption: All hospitals that would adopt MSL, adopt RSL before 2022 • Assumption: All hospitals have a similar share in BC patients to calculate the number of hospitals per technique • Assumption: A new localization technology has a redemption period of 5 years (implementation costs)													
	**localization technique used in % patients per year** *(# of hospitals that use a certain technology)*												
	**WGL**	**RSL**						**MSL**					
2017	79% (83)	21% (22)						0% (0)				[20]	
2018	71% (74)	29% (31)						0% (0)				Adoption curve of Rogers	
2019	60% (63)	40% (42)						0% (0)				Adoption curve of Rogers	
2020	52% (55)	48% (50)						0% (0)				Adoption curve of Rogers	
2021	40% (42)	60% (63)						0% (0)				Adoption curve of Rogers	
2022	30% (31)	70% (74)						0% (0)				Assumption	
				
*Standard of care with the introduction of MSL* • Assumption: Adoption follows the adoption curve of Rogers [28] • Assumption: All hospitals have a similar share in BC patients to calculate the number of hospitals per technology • Assumption: A new localization technique has a redemption period of 5 years (implementation costs)													
	**localization technique used in % patients per year** *(# of hospitals that use a certain technology)*												
	**WGL**		**RSL**						**MSL**				
2017	79% *(83)*		21% *(22)*						0% *(0)*			[20]	
2018	76% *(80)*		24% *(25)*						0% *(0)*			Adoption curve of Rogers	
2019	72% *(76)*		27% *(28)*						1% *(1)*			Adoption curve of Rogers	
2020	65% *(68)*		32% *(34)*						3% *(3)*			Adoption curve of Rogers	
2021	53% *(56)*		36% *(38)*						11% *(11)*			Adoption curve of Rogers	
2022	30% *(31)*		40% *(42)*						30% *(32)*			Expert opinion NKI-AvL	
**Intervention costs**													
**WGL**					**€ 2 617**								
Personnel costs						€279				*(11%)*		[29,30]	
Material costs						€43				*(2%)*		Hospital specific purchase costs (interviews)	
Intervention costs (surgery and imaging)						€2 173				*(83%)*		[31] and NKI-AvL	
Equipment costs						-				*(0%)*			
Overhead						€ 123				*(5%)*		[30]	
**RSL**					**€ 2 834**								
Personnel costs						€321				*(11%)*		[29,30]	
Material costs						€118				*(4%)*		Hospital specific purchase costs (interviews)	
Intervention costs (surgery and imaging)						€2 173				*(77%)*		[31] and NKI-AvL	
Equipment costs						€53				*(2%)*		Hospital specific purchase costs (interviews)	
Overhead						€168				*(6%)*		[30]	
**MSL**					**€ 2 662**							without the costs of the magnetic seed	
Personnel costs						€279				*(9%)*		[29,30]	
Material costs						€12				*(17%)*		Hospital specific purchase costs (interviews)	
Intervention costs (surgery and imaging)						€2 173				*(73%)*		[31] and NKI-AvL	
Equipment costs						€49				*(2%)*		Hospital specific purchase costs (interviews)	
Overhead						€149				*(5%)*		[30]	
Costs of the magnetic seed					+	€100 – €500						Assumption	
**Additional costs for patients receiving neoadjuvant chemotherapy**													
Using WGL (material and overhead)						€146						Hospital specific purchase costs (interviews); [30]	
Using MSL (material and overhead)						€146							
Average inflation ratio to account for an increase in costs in the future						1.0116						Assumption based on Dutch inflation rates of the past 5 years [32]	
**Implementation costs**													
**WGL**					N.A.								
**RSL** *(yearly costs)*					**€ 26 826** *(€5*.*553)*							Based on costs of 2017 [29,30] and NKI-AvL Process analysis by interviews in 5 hospitals. On average 332.75 hours of work	
Personnel						€18 629							
Overhead						€8 197							
**MSL** *(yearly costs)*					**€ 2 794** *(€578)*							Based on costs of 2017 [29,30] and NKI-AvL Process estimation based on interviews in the NKI-AvL estimated hours of work: 24 for training and writing protocols	
Personnel						€1 940							
Overhead						€854							

**Abbreviations:** WGL: Wire-guided Localization, MSL: Magnetic Seed Localization, RSL: Radio-active Seed Localization, BC: Breast Cancer S2 Appendix and C contain specific details on the cost components incorporated in the intervention costs (including actual costs) presented here.

#### 2.2.2. Expected utilization of localization techniques.

In 2017, RSL and WGL were used in respectively 21% and 79% of the BC patients, due to hospital differences [20]. To simulate future uptake, we assumed that WGL is not implemented in the coming years but that RSL or MSL will be implemented as a new technology in the future.

The potential future uptake of RSL and MSL in 2022 was estimated by experts working in the Netherlands Cancer Institute (NKI) where MSL is used in a research setting next to RSL [15]. Since, theoretical models describe that having knowledge on the innovation and the degree of relative advantage are important factors in the adoption decision, we did not consider it likely that very fast implementation would occur [28,33]. The uptake of MSL was estimated to be 30% in 2022 and the total usage of RSL and WGL 40% and 30% respectively. For SoC in 2022, the uptake of RSL and WGL was estimated at 70% and 30% respectively, assuming that hospitals wiling to adopt MSL (30%) adopt RSL instead.

**Fig 2:**
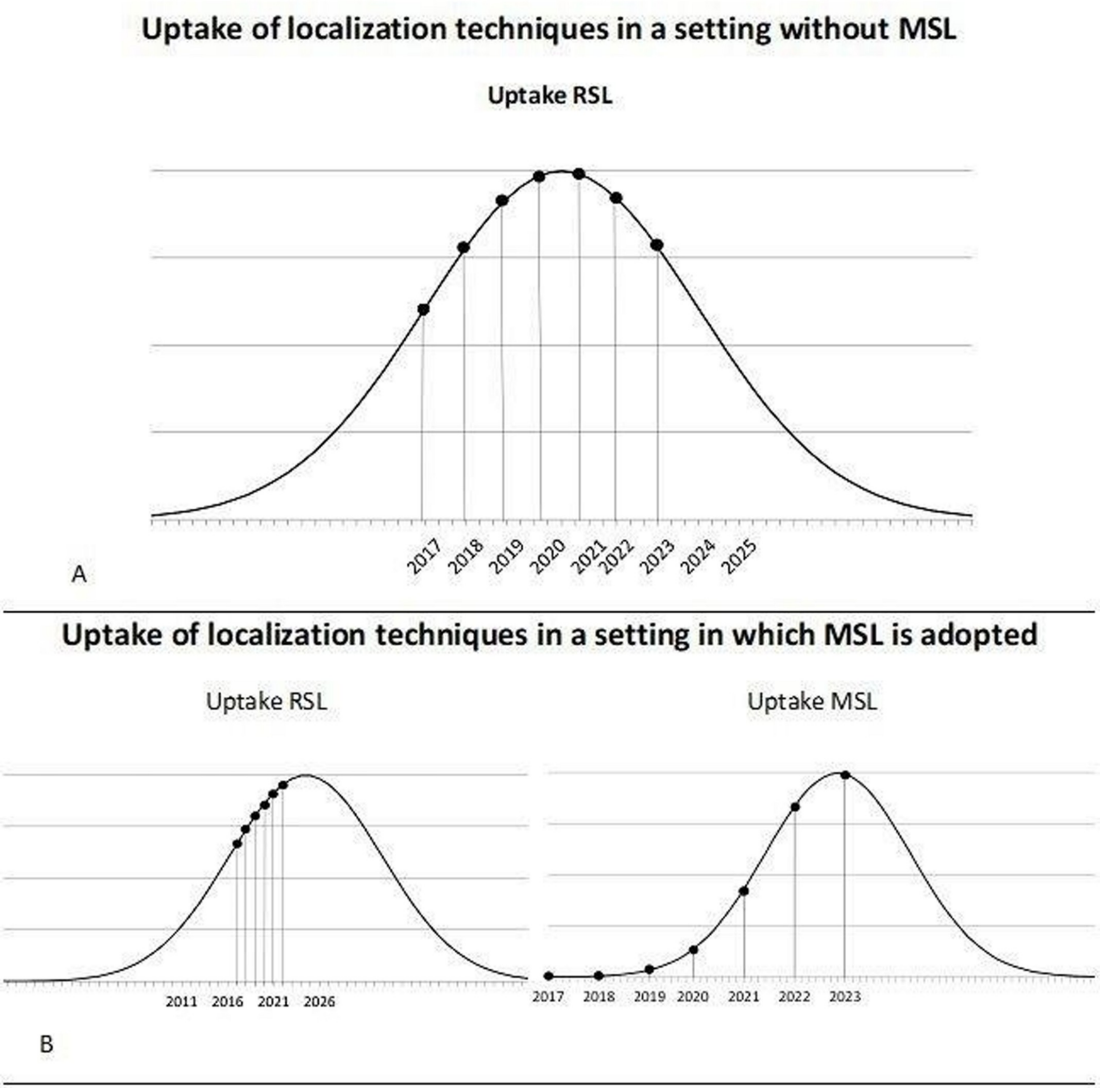
The expected uptake of RSL and MSL for both Standard of Care (SoC) and SoC with the adoption of MSL. (A) Shows the uptake of RSL when MSL is not implemented based on the adoption curve of Rogers and the assumed uptake of localization techniques in 2022: 70% RSL, 30% WGL. (B) Shows the uptake curves for RSL and MSL based on the adoption curve of Rogers and the assumed uptake of localization techniques in 2022: 30% MSL, 40% RSL. These curves were used to identify the number of patients per year receiving one of the technologies and to identify the hospitals that transfer from one technology to another. Abbreviations: WGL = wire-guided localization, RSL = radioactive seed localization, MSL = magnetic seed localization.

The classic diffusion theory by Rogers was used to estimate the adoption speed of RSL and MSL [28,34]. The annual uptake of MSL and RSL was estimated by using the “S”-shaped curve proposed by Rogers. This is shown in Fig 2 and Table 1. According to the projected diffusion curves, the adoption rates for 2023 were estimated to allocate the implementation costs of RSL and MSL in 2022.

#### 2.2.3 Intervention and implementation costs.

In the Netherlands, reimbursement for the use of localization techniques during breast cancer surgery is part of a budget allocated for a specific combination of diagnosis and treatment. Therefore, specific costs for using a localization technology apart from e.g. hospital stay, are not specified. By means of Activity Based Costing (ABC) costs for using the localization technologies were estimated. This method takes into account all activities consumed within a process and allocates costs to the resources required for these activities [35].

*Clinical process per localization technology*. First the processes had to be drafted for using WGL, RSL and MSL. The processes were evaluated by clinical expert interviews in eight hospitals in 2017 (five using RSL, three using WGL), “real-time” observations, literature and hospital treatment protocols. Since MSL was only used in one Dutch hospital, the MSL process was based on interviews held in that institute (NKI-AvL). The expert interviews assessed also the implementation process for RSL (evaluated in five hospitals) and MSL (evaluated in the NKI-AvL only). S1 Appendix shows the workflow per localization technology.

In drafting the processes, the incidents associated with the use of radioactive seeds were included in the process. Based on literature, the duration of seed placement (45 min) and surgery (90 min) were assumed to be similar between the localization techniques [4,12] and migration of magnetic seeds was assumed to be negligible [15,23]. Furthermore, based on multiple studies comparing WGL and RSL, minor complications e.g. wound infection, and displacement of the wire or seed were neglected as they were assumed to be uncommon and equal for the three localization technologies [10,12,36]. For the implementation processes we evaluated the numbers of staff involved and their number of hours invested in processes as: drafting protocols, performing a risk analysis, training, obtaining a license and internal procedures.

*Cost calculation*. To calculate the costs of each process step, the Dutch manual for cost calculations was used [30]. Personnel costs were calculated by multiplying the reference costs or gross salaries according to the collective agreement for hospitals of 2017 to the amount of time a staff member was occupied per process step [29]. Those costs were also used to calculate the implementation costs by multiplying the costs for the involved staff and the number of hours spend for implementation. The costs for surgery, pathology assessment and seed/wire placement were based on internal hospital prices or regulated tariffs from the Dutch Healthcare Authority [31]. The materials used and costs of materials were based on data from the eight selected hospitals. The material costs of MSL incorporated: the non-magnetic polymer surgical tools (Blunt retractor, sharp Weitlaner, retractor and a small or long forceps [15]), sterile cover for the probe, and magnetic probes. The costs were based on hospital data and expert interviews in the NKI (Table 1 and S3 Appendix). For the costs of the polymer surgical tools, the average usage of the different tools was estimated (e.g. 50% for the blunt retractor). These values were multiplied by the prices of the tools (Internal cost information NKI-AVL). The magnetic seeds costs were based on list prices of two companies selling products for MSL and was included as a range between €100 and €500.

Although the equipment used in WGL and RSL were already bought and will be used for several procedures, we included the equipment costs to have a fair comparison to MSL. Equipment costs for RSL, WGL and MSL were based on actual acquisition costs from the participating hospitals. Since the gamma probe and contamination monitors, essential for using RSL, are also used in other procedures these costs were partly taken into account: 50% and 30% respectively. Finally, overhead was calculated over all costs except over the intervention and material costs to avoid double counting, using a general percentage of 44% [30].

The intervention costs included in the BIA model were: €2617, €2834, €2662 (without magnetic seed) for WGL, RSL and MSL respectively. The additional costs per patient receiving NACT in WGL and MSL were €146, and the implementation costs for MSL and RSL were: €2794 and €26826 respectively. These costs and details on the analysis are presented in Table 1 and S2 and S3 Appendixes.

### 2.3 Analysis

To perform the analysis, Microsoft Excel version 2010 was used. The BIA compares the total intervention costs of the localization technologies used per year plus the yearly implementation costs of the hospitals that are expected to transfer to a different technology for both SoC and SoC with MSL. To calculate the yearly treatment costs, the yearly BC incidence was multiplied by the percentage of patients receiving localization during surgery and by the yearly uptake percentages of the localization technologies. These numbers were multiplied by the costs per localization technology including the additional costs for the proportion of patients receiving NACT. The future costs for 2018 and later were corrected using an average inflation ratio based on the Dutch inflation ratios of the previous five years [32].

### 2.4 Sensitivity analysis

The model structure and input parameters were based on several assumptions and therefore associated with a level of uncertainty. To evaluate the impact of our assumptions, deterministic sensitivity analyses (DSA) were conducted.

First, a one-way sensitivity analysis was conducted on the results of the cost analysis for RSL and MSL to identify the parameters with the highest influence. Upper and lower limits of 20% were used varying the for instance the number of patients per hospital, equipment costs and duration of placing the marker. Second, a one-way sensitivity analysis was conducted on the BIA results for the year 2022 with fixed magnetic seed costs of €200. Also upper and lower limits of 20% were used to check the influence of several input parameters. For example: implementation costs, treatment costs, and the percentage of patients receiving NACT. Finally, three alternative diffusion estimates were tested: a constant uptake of MSL, changing the adoption speed of MSL (slower, faster), and changing the initial uptake of RSL in 2017 to (1) 30% RSL, 70%WGL and (2) 40% RSL and 60% WGL.

### 2.5 Compliance with ethical standards

All procedures performed in studies involving human participants were in accordance with the ethical standards of the institutional and/or national research committee and with the 1964 Helsinki declaration and its later amendments or comparable ethical standards. For all the interviewees (employees of the eight hospitals involved in our research) we asked their permission to record the interviews and additionally we checked the information retrieved from the interview with each interviewee.

## Results

### 3.1 Budget impact analysis

**Fig 3:**
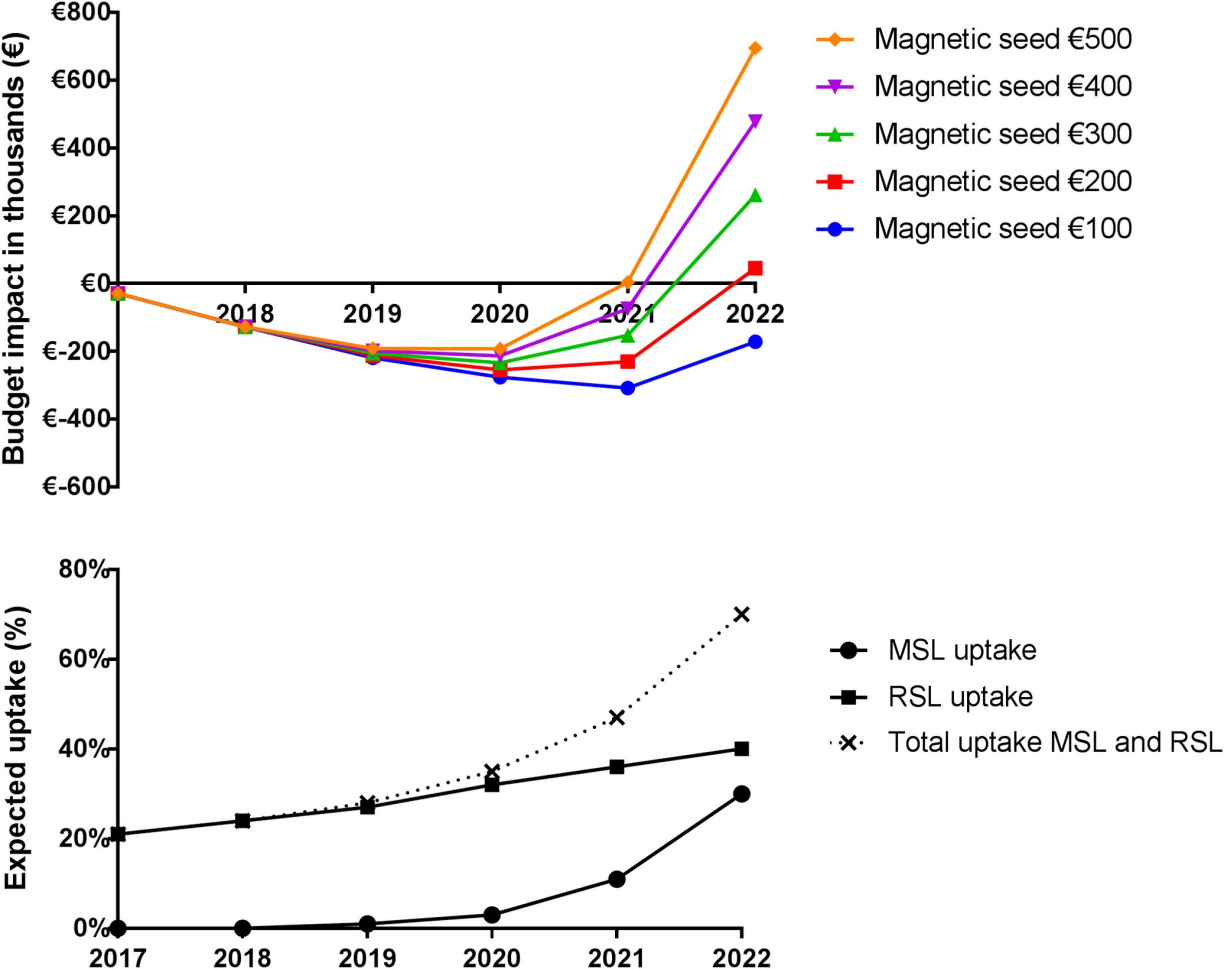
Total annual budget impact in respect of the uptake of RSL and MSL when MSL is adopted. The annual budget impact of a setting in which MSL is adopted compared to standard of care is visualized. In addition the expected uptake of RSL and MSL for the future situation is visualized as this explains the rise in budget impact. When a small percentage of hospitals is transferring to MSL instead of RSL e.g. year 2020 and 2021 and the cost of the magnetic seeds is ≤€200 a benefit is seen due to the smaller yearly implementation costs. This effect is overruled when more hospitals are transferring to MSL as the costs per patient are higher for MSL than for RSL. Abbreviations, WGL = wire-guided localization, RSL = radioactive seed localization, MSL = magnetic seed localization.

The results of the BIA model are shown in Table 2 and Fig 3. Total costs for SoC with RSL and WGL use increased from €14.7m in 2017 to €16.9m in 2022 due to an increased number of BC patients and increased number of hospitals implementing RSL. When MSL is increasingly adopted and the magnetic seed would only cost €100, total costs increased from €14.7m to €16.7m resulting in a BI of -€0.2m in 2022. With a magnetic seed price of €500, total healthcare costs increased from €14.7m to €17.6m, resulting in a BI of €0.7m in 2022. At a price level of €178 for the magnetic seed, the BI in 2022 is neutral.

**Table 2:** Base case results of the budget impact analysis of adopting magnetic seed localization (MSL) in breast conserving surgery.

	**2017**	**2018**	**2019**	**2020**	**2021**	**2022**
# BC patients that are being localized	5472	5504	5536	5568	5600	5633
# patients receiving NACT	821	826	830	835	840	845
**Standard of care (without MSL)**						
# hospitals using WGL	83	75	63	55	42	31
# hospitals using RSL	22	30	42	50	63	74
# hospitals using MSL	0	0	0	0	0	0
Patients localized with WGL (NACT)	4323 (821)	3908 (793)	3322 (717)	2895 (663)	2240 (542)	1690 (431)
Patients localized with RSL (NACT)	1149 (218)	1596 (324)	2214 (478)	2673 (612)	3360 (813)	3943 (1005)
Patients localized with MSL (NACT)	0 (0)	0 (0)	0 (0)	0 (0)	0 (0)	0 (0)
Healthcare costs (€)	14,689,668	15,031,576	15,412,565	15,769,062	16,177,124	16,571,516
Implementation costs (€)	46,647	111,532	159,268	231,702	292,765	309,402
** Total (€)**	**14,736,316**	**5,143,108**	**15,571,833**	**16,000,765**	**16,469,889**	**16,880,918**
**Standard of care with adoption of MSL**						
# hospitals using WGL	83	80	76	68	56	31
# hospitals using RSL	22	25	28	34	38	42
# hospitals using MSL	0	0	1	3	12	32
Patients localized with WGL (NACT)	4323 (821)	4183 (849)	3986 (861)	3619 (829)	2968 (718)	1690 (431)
Patients localized with RSL (NACT)	1149 (218)	1321 (268)	1495 (323)	1782 (408)	2016 (488)	2253 (575)
Patients localized with MSL (NACT)	0 (0)	0 (0)	55 (12)	167 (38)	616 (149)	1690 (431)
Healthcare costs (€) when the magnetic seed costs:						
€ 100	14,689,668	14,987,005	15,298,487	15,645,802	16,046,702	16,564,325
€ 200	14,689,668	14,987,005	15,305,342	15,666,726	16,124,763	16,780,924
€ 300	14,689,668	14,987,005	15,312,197	15,687,651	16,202,824	16,997,522
€ 400	14,689,668	14,987,005	15,319,052	15,708,575	16,280,885	17,214,121
€ 500	14,689,668	14,987,005	15,325,907	15,729,499	16,358,945	17,430,720
Implementation costs (€)	17,493	35,803	66,881	96,055	132,563	162,039
**Budget impact (€)**	**2017**	**2018**	**2019**	**2020**	**2021**	**2022**
when the magnetic seed costs:						
€ 100	-29,155	-127,828	-219,411	-276,032	-308,493	-171,598
€ 200	-29,155	-127,828	-212,556	-255,107	-230,432	45,001
€ 300	-29,155	-127,828	-205,701	-234,183	-152,371	261,600
€ 400	-29,155	-127,828	-198,846	-213,259	-74,310	478,199
€ 500	-29,155	-127,828	-191,991	-192,334	3,750	694,798
BI in healthcare costs only (€) when the magnetic seed costs:						
€ 100	0	-52,099	-127,024	-140,385	-148,292	-24,235
€ 200	0	-52,099	-120,169	-119,460	-70,231	192,364
€ 300	0	-52,099	-113,314	-98,536	7,830	408,962
€ 400	0	-52,099	-106,459	-77,611	85,891	625,561
€ 500	0	-52,099	-99,604	-56,687	163,952	842,160

**Abbreviations:** WGL: Wire-guided Localization, MSL: Magnetic Seed Localization, RSL: Radio-active Seed Localization, BC: Breast Cancer, NACT: Neoadjuvant chemotherapy. All values are rounded.

Fig 3 shows that there is a benefit to adopt MSL due to the lower implementation costs. However, when more hospitals are implementing MSL and the intervention costs of using MSL are higher than for RSL and/or the percentage of NACT patients is increasing, the use of RSL and WGL are more cost-efficient for the Netherlands overall. For each hospital, which localization technology is most cost-efficient depends on the number of BC patients per year, proportion of patients receiving NACT and the current implemented localization technique. S4 Appendix contains an adjustable version of the BIA model to enable evaluation of the adoption of MSL for a different country or a hospital setting.

### 3.2 Sensitivity analysis

**Fig 4:**
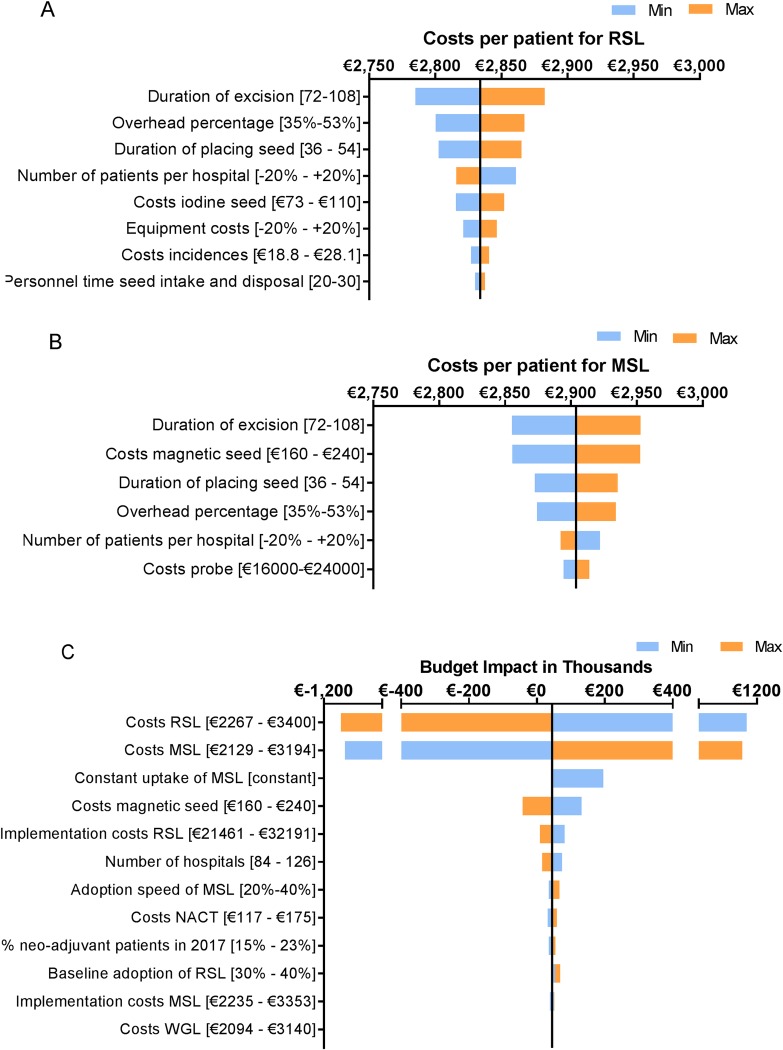
Results of the one-way sensitivity analyses. A. shows the results of the sensitivity analysis on the cost analysis results for RSL. The base case value is: 2,833.95. B. shows the results of the sensitivity analysis on the cost analysis results for MSL. The base case value is: 2,904.06 with a magnetic seed price of €200. C. shows the results of the sensitivity analysis on the results of the budget impact analysis in 2022, with a magnetic seed price of €200. The base case value is €45,00. Explanation regarding parameter “constant uptake of MSL”: The yearly uptake of WGL was hold constant and the uptake of RSL was linked to the uptake of MSL (RSL yearly uptake = 100%—%MSL—%WGL). Abbreviations: WGL = wire-guided localization, RSL = radioactive seed localization, MSL = magnetic seed localization.

The duration of the excision and seed placement, the costs of the magnetic seed and the overhead percentage drove the intervention costs of RSL and MSL the most (Fig 4A and 4B). Uncertainty in those parameters could have a great impact on the calculated costs per patient and thus on the results of the BIA. As Fig 4C demonstrates, the intervention costs had a substantial influence on the BIA results.

The different diffusion estimates incorporated in the DSA had a small impact on the budget impact (Fig 4C). A constant uptake of MSL showed an increased BI because the uptake of RSL in 2023 is then much higher than in the base case situation which results in higher implementation costs accounted in 2022 for the situation with MSL adoption. A steeper adoption curve of MSL showed an increased BI because the intervention costs of MSL are higher than those for RSL. These higher costs were not resolved by the lower implementation costs for MSL. The increased uptake of RSL in 2017 showed an increased BI, because the endpoint in 2022 for RSL was kept the same, and therefore less hospitals transferred to RSL per year in both scenarios resulting in lower total implementation costs especially for usual care.

## Discussion

The results of the BIA indicated that adoption of MSL in the Dutch healthcare system could be cost-saving due to the lower implementation costs for MSL (€2974) compared to RSL (€26826). However, to maintain this advantage after implementation phase: (1) the costs of using MSL per patient should not be substantially higher than those for RSL or (2) response monitoring with MRI should be enabled when using MSL in NACT patients or (3) the use of MSL should result in improved clinical outcomes compared to WGL and RSL.

To the best of our knowledge this is the first BIA on localization techniques in which the intervention costs of localization techniques have been evaluated in detail, including the additional activities related to using radioactive material. The results of the cost analysis could inform the decision to transfer from WGL to either MSL or RSL on a national or hospital level. The decision to adopt one of the technologies may be further supported by additional factors such as the improved resource allocation and impact of a localization method on logistics [3,4,6]. Besides, the MSL use could be used relatively easy outside the breast cancer indication, whereas the expansion of indications for RSL involves a time-consuming regulative route because of its radioactive nature [15,16]. These additional factors are important to take into account when deciding on the adoption of MSL but a detailed evaluation was out of the scope of this study.

The BIA results are mainly driven by the intervention costs (Fig 4). The costs used in our analysis are based on several Dutch hospitals. Since, country specific regulations related to safety of radioactive material can have an influence on the costs, the generalizability of the results from the cost-analysis to non-European countries is limited. Comparing our results to current evidence showed that the comparability of publications is limited because of variations in design choices, limited access to cost data, and differences in costs and materials used across countries [37–39]. Comparable studies included for instance re-excision rates, complication rates and cost savings related to logistics which resulted in overall savings for RSL compared to WGL [39–41]. Also, the presented costs are often relative differences instead of absolute numbers [39,40]. A study from a US perspective showed that RSL was also associated with higher material and personnel costs compared to WGL, but further validation of our results was limited as the results were presented as savings per patient ($115) [39]. In general for using RSL in the US, our results are expected to underestimate the costs and budget impact as the procedures related to radioactive material use are different. Especially, regarding the allowed duration of iodine seeds to remain in situ (max of 5–7 days) implying that in NACT treated patients an additional marker has to be placed for response monitoring [39,42]. As in our analysis the additional marker placement is the main disadvantage of MSL compared to RSL, this would have a significant impact on our results and conclusion (budget impact of €-21,900 in 2022 in favor of MSL (magnetic seed = €200).

The main strength of the current analysis is the detailed insight in the costs of all three localization techniques, based on data from 8 Dutch hospitals. Our results can be used and adjusted on a hospital and country level to guide the decision to adopt RSL or MSL using the general model (S4 Appendix). As we were not able to include all available techniques in this field due to lack of detailed data, the model allows to include other promising alternatives to MSL such as Radioguided occult lesion localization [40,43], radar technology (SAVI SCOUT) [44,45] or Ultra Sound [46,47] applications. We have not been able to compare the results from our analysis to alternatives such as SAVI SCOUT, that recently received $510k approval from the FDA. As a trial is still to be reported upon (NCT03015649), we advise to perform a comparable analysis once the technology proves to have equal or better clinical value compared to existing technologies. Another strength of our analysis is the inclusion of the implementation costs to clarify the relation between the acceptable higher treatment costs but a less labor intensive implementation process compared to RSL.

The main limitations in this study are the assumptions regarding uptake of various techniques for 2022, and the early stage of our analysis. As the present analysis evaluates a new technology still in development and subject of clinical trials, the results from the cost-analysis related to MSL (treatment and implementation costs) are uncertain and of potentially limited applicability. The impact of the implementation costs on the budget for 2017 to 2022 could be underestimated, due to allocation of the implementation costs over 5 years and the majority of hospitals were assumed to adopt a new technology in the final two years. Other limitations were: (1) the selection of the hospitals, as this could have biased the cost-analysis results. Although we incorporated all types of hospitals (academic, general and specialized) and hospitals located in different areas of the country, this could limit the generalizability of the budget impact analysis. (2) Not being able to incorporate the logistical hurdles when using WGL and therefore the intervention costs of WGL were underestimated. This however would not have altered the conclusions as the benefits are similar for MSL and RSL compared to WGL. A final limitation (3) is the main assumption that the efficacy of MSL and RSL is similar to WGL. Future comparative studies should verify whether this is truly the case. If clinical benefit is expected these factors should be incorporated in this analysis or a cost-effectiveness analysis should be performed.

## Conclusion

Our present analysis shows that MSL could be a new cost-efficient localization technology in guiding resections of non-palpable breast cancer tumors in the Netherlands. When the costs to use MSL are significantly higher than those for using RSL and WGL, the lower implementation costs for MSL will not outbalance these higher intervention costs. Manufactures should consider cost-effectiveness when determining retail price of the magnetic seed.

## Supporting information

S1 Appendix Flow diagrams of included steps in the ABC analyses of WGL, RSL and the MSL. (DOCX)

S2 Appendix Detailed description of intervention costs. (DOCX)

S3 Appendix Overview of the included materials and activities. (DOCX)

S4 Appendix (XLSX)

## List of abbreviations

ABC: Activity-Based Costing
BC: Breast Cancer
BCS: Breast-Conserving Surgery
BI: Budget Impact
BIA: Budget Impact Analysis
DCIS: Ductal Carcinoma In Situ
MSL: Magnetic seed localization
MRI: Magnetic Resonance Imaging
NKI: Netherlands Cancer Institute (*Nederlands Kanker Instituut)*
OR: Operating Room
RSL: Radioactive Seed Localization
WGL: Wire-Guided Localization
