# Supplementary figures and images for: Early budget impact analysis on magnetic seed localization for non-palpable breast cancer surgery
Source: PLoS One. 2020 May 13;15(5):e0232690. doi: 10.1371/journal.pone.0232690 (PMC7219736; doi:10.1371/journal.pone.0232690)

**Appendix A: Flow diagrams of included steps in the ABC analyses of WGL, RSL and the MSL**


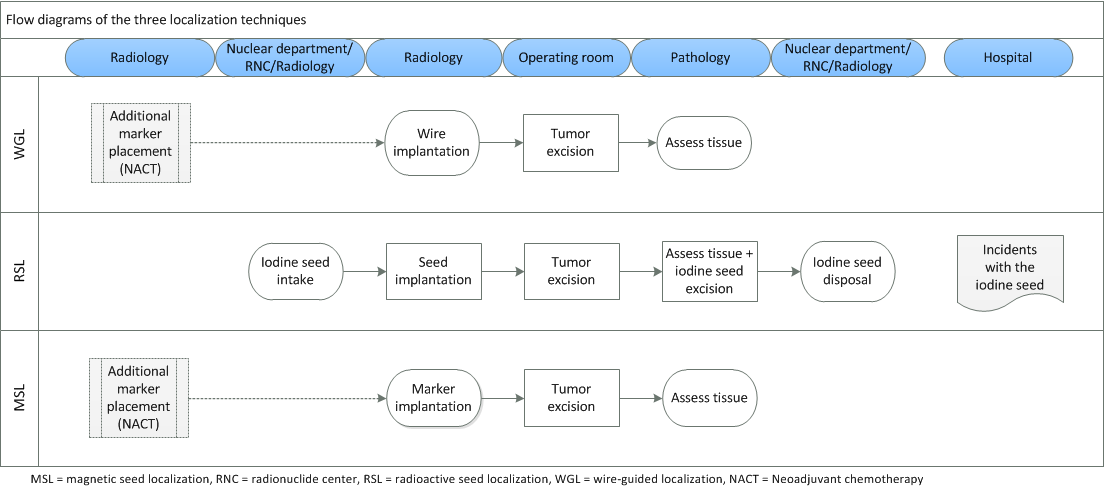

Supplement: S1 Appendix — (DOCX) [file pone.0232690.s001.docx]
