# Supplementary material for: Early budget impact analysis on magnetic seed localization for non-palpable breast cancer surgery
Source: PLoS One. 2020 May 13;15(5):e0232690. doi: 10.1371/journal.pone.0232690 (PMC7219736; doi:10.1371/journal.pone.0232690)
Supplement: S2 Appendix — (DOCX) [file pone.0232690.s002.docx]

**Appendix B – detailed description of intervention costs**

| Step | Cost category | | WGL | RSL | MSL | Source of cost |
| --- | --- | --- | --- | --- | --- | --- |
| *Neo-adjuvant* | *Material* | | *€102* |  | *€102* | Interviews |
|  | *Overhead* | | *€45* |  | *€45* | ^26^ |
| Seed intake | Personnel | |  | €8 |  | ^26,27^ |
|  | Overhead | |  | €3 |  | ^26^ |
| Implantation of seed, wire, marker | Personnel | | €109 | €109 | €109 | ^26,27^ |
|  | Material | | €43 | €111 | €0 | Interviews |
|  | Intervention | | €179 | €179 | €179 | ^28^ |
|  | Equipment | |  | €1 |  | Interviews |
|  | Overhead | | €48 | €48 | €48 | ^26^ |
| Tumor excision guided by seed or wire | Personnel | | €170 | €170 | €170 | ^26^ |
|  | Material | |  | €7 | €12 | Procurement NKI |
|  | Intervention | | €1,329 | €1,329 | €1,329 | NKI |
|  | Equipment | |  | € 29 | € 49 | Interviews |
|  | Overhead | | €75 | €90 | €101 | ^26^ |
| Assessment of tumor and seed excision | Personnel | |  | €6 |  | ^26,27^ |
|  | Intervention | | €666 | €666 | €666 | ^28^ |
|  | Equipment | |  | €22 |  | Interviews |
|  | Overhead | |  | €12 |  | ^26^ |
| Seed disposal | Personnel | |  | €5 |  | ^26,27^ |
|  | Overhead | |  | €2 |  | ^26^ |
| Incidents | Personnel | |  | €23 |  | ^26,27^ |
|  | Equipment | |  | €2 |  | ^26^ |
|  | Overhead | |  | € 11 |  | ^26^ |
| Implementation: training  Implementation: Protocol drafting  Implementation: Obtaining license  Implementation: Risk analysis  Implementation: Internal procedures | Personnel  Personnel  Personnel  Personnel  Personnel | | N/A  N/A  N/A  N/A  N/A | €4.321  €4.290  € 2.224  € 2.972  € 6.430 | €1.189  €751  N/A  N/A  N/A | ^26,27^  ^26,27^  ^26,27^  ^26,27^  ^26,27^ |
| Total implementation costs*  Overhead costs | Personnel  Overhead | | N/A | €18.629  €8.197 | €1.940  €854 | ^26,27^  ^26^ |
| Summary | | | | | | |
| Average cost per patient | | €2.617 | | €2.834 | €2.662 |  |
| Variable extra cost per neo-adjuvant patient | | €147 | | €0 | €147 |  |
| Fixed implementation costs | | N/A | | €26,826 | €2,794 |  |

**Appendix B: Disaggregated and summarized costs per localization technique, process step and cost category**

Material and equipment costs are including VAT. Implementation costs are shown as fixed costs. Other costs are shown as cost per patient. The average cost per patient does not include implementation costs and the additional costs for the neo-adjuvant setting. MSL= Magnetic Seed Localization, NKI = Netherlands cancer institute, NZA = Dutch healthcare authority, RSL = radioactive seed localization, WGL = wire-guided localization, ‘x’= no personnel, material or equipment costs are needed, N/A = not applicable. * the total costs are not corresponding with the sum of the averages per process step as the total average costs were calculated over the total implementation costs per hospital.
