# Supplementary material for: Early budget impact analysis on magnetic seed localization for non-palpable breast cancer surgery
Source: PLoS One. 2020 May 13;15(5):e0232690. doi: 10.1371/journal.pone.0232690 (PMC7219736; doi:10.1371/journal.pone.0232690)
Supplement: S3 Appendix — (DOCX) [file pone.0232690.s003.docx]

**Appendix C – Overview of the included materials and activities**

| **Step** | **Cost category** | **WGL** | **RSL** | **MSL** |
| --- | --- | --- | --- | --- |
| ***Neo-adjuvant*** | *Material* | Marker or clip | - | Marker or clip |
| **Seed intake** | *Personnel* | - | RNC assistant/radiation expert/nuclear medicine staff for 15 minutes | - |
| **Seed/wire/marker implantation** | *Personnel* | Radio diagnostic laboratory worker and a radiologist for 45 minutes | Radio diagnostic laboratory worker and radiologist for 45 minutes | Radio diagnostic laboratory worker and radiologist for 45 minutes |
|  | *Material* | Wire incl. needle  Anesthesia | I-125 seed  Needle  Materials for preparing seed  Anesthesia | Pre-loaded applicator with magnetic marker  Anesthesia |
|  | *Intervention* | Ultrasound*  Mammography | Ultrasound*  Mammography | Ultrasound*  Mammography |
|  | *Equipment* |  | Radiation detector |  |
| **Tumor excision guided by seed/wire/marker** | *Personnel* | Surgeon (assumed duration of surgery of 90 minutes) | Surgeon (assumed duration of surgery of 90 minutes) | Surgeon (assumed duration of surgery of 90 minutes) |
|  | *Material* |  | Probe cover | Probe cover  Polymer surgical instruments |
|  | *Intervention* | Operation | Operation | Operation |
|  | *Equipment* |  | Gamma probe | Magnetic probe |
| **Assessment of tumor and seed excision** | *Personnel* |  | Analyst for 12.5 minutes on average |  |
|  | *Intervention* | Complex resection | Complex resection | Complex resection |
|  | *Equipment* |  | Radiation detector |  |
| **Seed disposal** | *Personnel* | - | RNC assistant/radiation expert/nuclear medicine staff for 10 minutes | - |
| **Incidents** | *Personnel incident* |  | Radiation expert/ clinical physicist/ nuclear department for 16 hours |  |
|  | *Personnel monitoring* | - | Several people from the involved departments: radiology, surgery, pathology and nuclear department in total for an average of 42.5 hours per year | - |
|  | *Equipment* |  | Contamination monitor |  |
| **Implementation** | *Personnel* | - | Several people from the involved departments: radiology, surgery, pathology and nuclear department  On average this took in total 322.75 hours | Several people from the involved departments: radiology and surgery  Estimated to take 24 hours |

*All seeds/wires/markers were assumed to be implanted by ultrasound guidance, although a small percentage of implantations is guided through stereotactic guidance. MSL = magnetic marker localization, RSL = radioactive seed localization, WGL = wire-guided localization.
